# Log interpretation method of resistivity low-contrast oil pays in Chang 8 tight sandstone of Huanxian area, Ordos Basin by support vector machine

**DOI:** 10.1038/s41598-022-04962-0

**Published:** 2022-01-20

**Authors:** Ze Bai, Maojin Tan, Yujiang Shi, Xingning Guan, Haibo Wu, Yanhui Huang

**Affiliations:** 1 School of Earth and Environment, Anhui University of Science & Technology, Huainan, 232001 China; 2 School of Geophysics and Information Technology of China University of Geosciences, Beijing, 100083 China; 3 A Research Institute of Exploration and Development, PetroChina Changqing Oilfield Company, Xi’an, 710018 China; 4 Institute of Energy, Hefei Comprehensive National Science Center, Hefei, 230071 Anhui Province China; 5 State Key Laboratory of Petroleum Resources and Prospecting, China University of Petroleum, Beijing, 102249 China

**Keywords:** Energy science and technology, Mathematics and computing

## Abstract

Resistivity low-contrast oil pays are a kind of unconventional oil resource with no obvious difference in physical and electrical properties from water layers, which makes it difficult to be identified based on the characteristics of the geophysical well logging response. In this study, the support vector machine (SVM) technology was used to interpret the resistivity low-contrast oil pays in Chang 8 tight sandstone reservoir of Huanxian area, Ordos Basin. First, the input data sequences of logging curves were selected by analyzing the relationship between reservoir fluid types and logging data. Then, the SVM classification model for fluid identification and SVR regression model for reservoir parameter prediction were constructed. Finally, these two models were applied to interpret the resistivity low-contrast oil pays in the study area. The application results show that the fluid recognition accuracy of the SVM classification model is higher than that of the logging cross plot method, back propagation neural network method and radial basis function neural network method. The calculation accuracy of permeability and water saturation predicted by the SVR regression model is higher than that based on the experimental fitting model, which indicates that it is feasible to carry out logging interpretation and evaluation of the resistivity low-contrast oil pays by the SVM method. The research results not only provide an important reference and basis for the review of old wells but also provide technical support for the exploration and development of new strata.

## Introduction

With the increasing volatility of international oil prices and the continuous reduction of oil reservoir scale, the resistivity low-contrast oil resources with strong concealment has received much interest in recent years. Carrying out the research on logging interpretation and evaluation method of resistivity low-contrast oil pays has become the most practical choice to supplement conventional oil resources and reduce oilfield exploration cost^1,2,27^. The resistivity low-contrast oil pay has the characteristics of little difference in porosity and resistivity logging response from water layer, and the oil saturation of resistivity low-contrast oil layer is relatively low^3,4^. At present, low porosity and low permeability reservoirs represented by tight sandstone has become the main battlefield to ensure the supply of oil and gas resources^5^. However, the complex pore structure and strong heterogeneity of tight sandstone reservoir reduce the sensitivity of the logging response to pore fluid, resulting in more resistivity low-contrast oil pays developed, and it is more difficult to interpret and identify this kind of reservoir by using conventional logging interpretation methods^6,7,25^.

In recent years, data mining technology has been increasingly applied in oil exploration and development, especially for unconventional reservoirs with unclear logging response characteristics, and how to use data mining technology to effectively solve some complex problems existing in the actual production of oil fields is of great significance^8–10^. Some classical optimization algorithms, such as the neural network method, support vector machine and fuzzy clustering method, provide a new technology for the identification of resistivity low-contrast oil pays^11,12^. Guo et al.^13^ predicted the water saturation at the lower limit of three water models by using the generalized neural network (GRNN) and particle swarm optimization support vector machine (PSO-SVM), which is in good agreement with the core analysis results in the Sulige tight sandstone reservoir. Chen and Peng^14^ used a BP neural network to train and learn the mathematical characteristics of logging curves of low resistivity oil reservoirs, which improved the accuracy of fluid identification and reservoir parameter prediction. Singh et al.^15^ used the stepwise linear regression, multilayer feed forward neural (MLFN) network method to predict the 2D distribution of P-wave velocity, resistivity, porosity, and gas hydrate saturation. Miah et al.^16^ used the multilayer perception artificial neural network (MLP-ANN) and kernel function-based least-squares support vector machine (LS-SVM) techniques to develop predictive models for water saturation, and the prediction performance was better than that of other models. Baouche and Nabawy^17^ applied the fuzzy logic technique that enabled a reservoir zonation of the Southern Hassi R'Mel Gas Field into several hydraulic flow units with various reservoir properties, and then the permeability values of each flow unit were predicted. With the deepening of research, many machine learning algorithms based on theoretical mathematics have been proposed, and each has its own advantages and disadvantages. However, the key to applying this kind of method to log interpretation of actual formation is to select appropriate training data as input^18,19^. In this study, the support vector machine (SVM) learning method based on VC dimension theory in statistical learning and the structural risk minimization principle (SRM) were used to establish the interpretation model. By analyzing the relationship between logging response and pore fluid, training data were optimized, and SVM classification model for fluid identification and support vector machine regression (SVR) model for reservoir parameter prediction were established. The application results show that the log interpretation models established by the SVM method are more effective than conventional method, which proves that it is feasible to identify and evaluate resistivity low-contrast oil pays based on SVM method.

## Geological and logging response characteristics of research area

**Figure 1:**
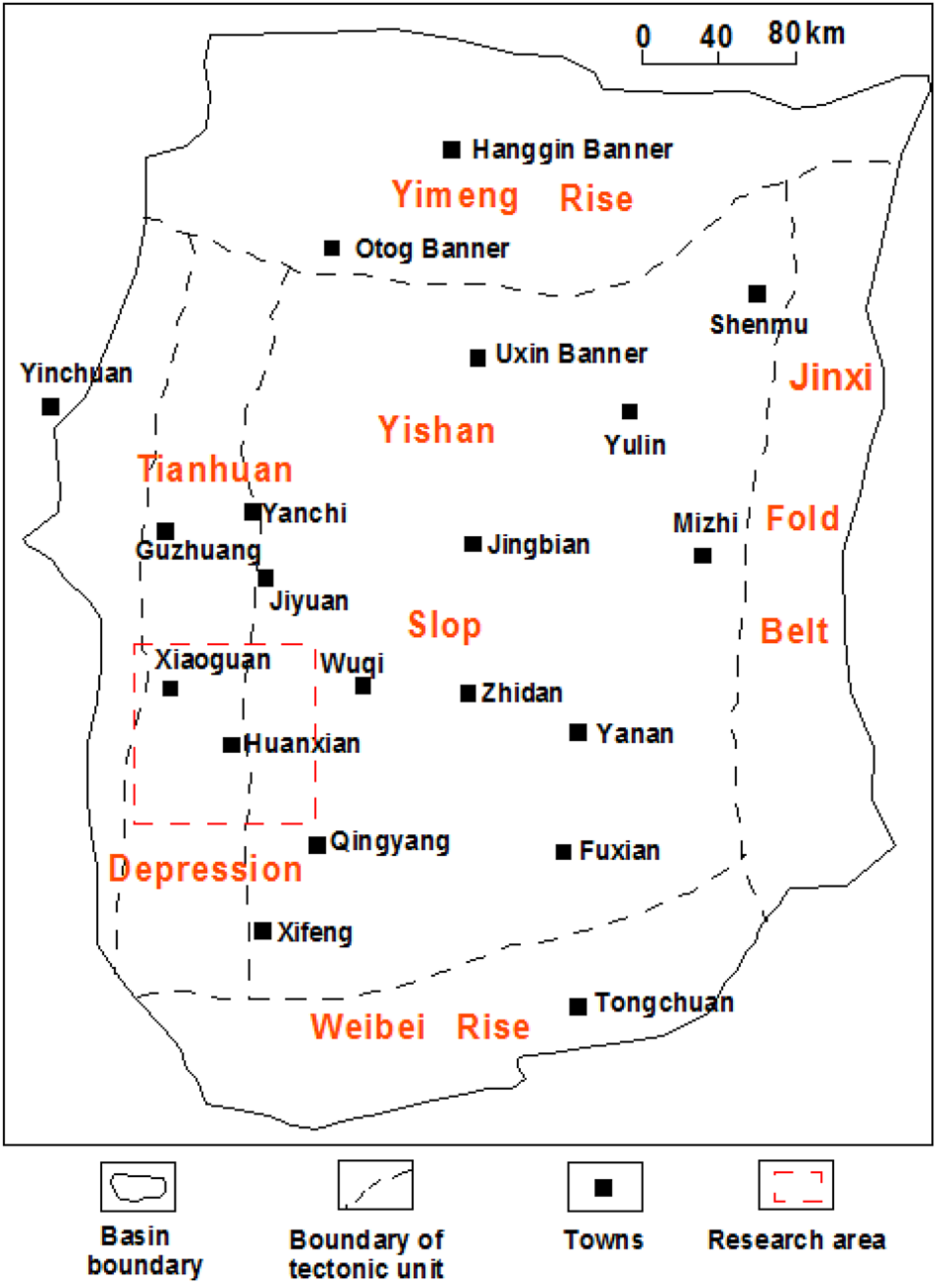
Geographical location of the research area.

The Ordos Basin is the second largest sedimentary basin in China, bearing more than half of China's energy output^20,21^. The Huanxian area is located in southwestern Ordos Basin, and the regional geological structure crosses the Tianhuan Depression and Yishan Slope from west to the east (Fig. 1). The Chang 8 member of the Yanchang Formation developed in the Huanxian area is a typical tight sandstone reservoir with large sedimentary thickness. The oil source of Chang 8 tight sandstone reservoir mainly comes from the overlying Chang 7 high-quality source rock, which makes it has great exploration and development potential^22,23^. However, with deepening of oil and gas exploration and development in this area, the problem of identification and evaluation of resistivity low-contrast oil reservoir has become increasingly prominent^7,24,25^.

**Figure 2:**
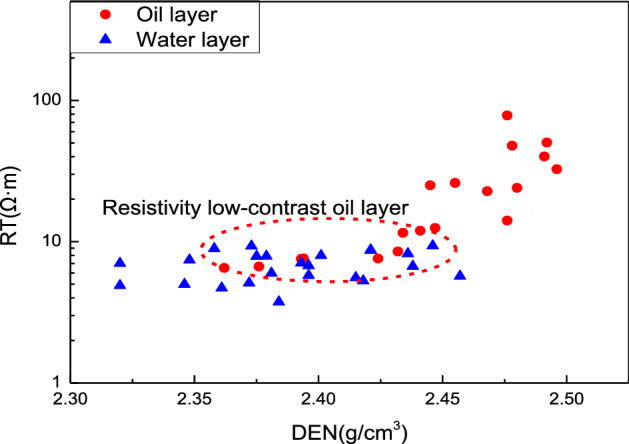
The cross plot of reservoir resistivity and density.

According to previous studies, the genesis of resistivity low-contrast oil reservoir is very complex, and it is usually caused by many factors^26,27^. Figure 2 shows the relationship between resistivity and density logging response of oil layer and water layer established by the oil test data in the study area. And the logging response characteristics of different fluids were shown in Table 1. It can be seen that the density (DEN) value and reservoir resistivity (RT) value of resistivity low-contrast oil reservoir is lower than that high resistivity oil reservoir. And the relative shale content ($\Delta$GR) has little difference between resistivity low-contrast oil reservoir and high resistivity oil reservoir, indicating that the shale content of reservoir has little effect on resistivity. In addition, the relative amplitude of spontaneous potential ($\Delta$SP) in resistivity low-contrast oil reservoir is higher than that of high resistivity oil reservoir, which reflects that the difference of formation water salinity property is likely to be an important reason for the change of electrical property. Besides, the complex pore structure and high irreducible water saturation in tight sandstone reservoir make it difficult for conventional logging to identify and evaluate resistivity low-contrast oil reservoir, which seriously restricts the exploration progress and development of oil resources in this area. Therefore, it is important to develop more effective methods to provide new logging technical support for the exploration and development of resistivity low-contrast oil layers.

**Table 1:** Logging response characteristics of different fluids.

Fluids	AC(us/m)		DEN(g/cm^3^)		ΔGR		ΔSP		RT(Ω.m)	
	Range	Ave	Range	Ave	Range	Ave	Range	Ave	Range	Ave
Resistivity low-contrast Oil	209–240	223	2.37–2.52	2.38	0.1–0.34	0.24	0.5–0.94	0.72	6.4–20.5	10.2
High resistivity Oil	212–231	221	2.44–2.52	2.48	0.09–0.45	0.26	0.4–0.85	0.66	23.03–92	57.68
Water	220–240	235	2.37–2.45	2.39	0.15–0.24	0.20	0.6–0.83	0.72	8.7–10.13	9.25

## Method and theory

Different from the neural network method to solve the number of hidden nodes of neurons, the basic idea of a support vector machine for reservoir parameter prediction is to map the input space to a high-dimensional space by introducing a kernel function and then solve a linearly separable hyperplane or function in this high-dimensional space, which can separate all data types in the original space. The greater the separation distance is, the better the classification effect. Finally, the nonlinear discrimination ability of the original spatial data is realized^28^.

Taking ${\text{T} = }\left\{ {{(}{\mathbf{x}}_{{\mathbf{i}}}, \text{y}_{\text{i}} {)}\left| {{\text{i} = 1,2,}\ldots,\text{n}} \right.} \right\}$ and ${\mathbf{x}}_{{\mathbf{i}}} \in R^{P}$ as the input data, where ${\mathbf{x}}_{{\mathbf{i}}}$ is the logging data related to the predicted parameters, and $\text{y}_{i}$ is the core analysis data, that is, the target value.

Suppose that in high-dimensional space, the hyperplane or line function that can separate the two types of samples satisfies:

1 $$g(\text{x}_{i} ) = \left\langle {{\mathbf{w}}_{ij}\cdot {\mathbf{x}}_{i} } \right\rangle + \text{b}_{ij}$$

where ${\mathbf{w}}_{ij}$ is the weight vector representing high-dimensional unknown coefficients and $\text{b}_{ij}$ is a constant term. To use function (1) to distinguish all input data samples without error, function $\text{y}_{\text{k}} {(}\left\langle {{\mathbf{w}}\cdot{\mathbf{x}}} \right\rangle { + \text{b}) - 1} \ge {0}$ should be satisfied. When the classification interval is maximum, function $\phi ({\mathbf{w}}) = \frac{{1}}{{2}}{\mathbf{w}}^{\text{T}} {\mathbf{w}}$ should be minimum. In this way, the problem of solving the optimal hyperplane in high-dimensional space is transformed into the minimum value problem of the following convex programming function:

2 $$\phi ({\mathbf{w}},\xi ) = \frac{{1}}{{2}}{\mathbf{w}}^{\text{T}} {\mathbf{w}}{ + \text{C}}\sum\limits_{{{\text{k} = 1}}}^{\text{n}} {\upxi _{\text{k}} }$$

Which satisfies the following constraint condition:

3 $$\text{y}_{\text{k}} {(}\left\langle {{\mathbf{w}}\cdot{\mathbf{x}}} \right\rangle { + \text{b})} \ge {1 - \xi }_{\text{k}},\,\, { \text{k} = 1,}\ldots,\text{n}$$

where $\xi_{k}$ is a nonnegative relaxation variable introduced when the sample data are linearly inseparable; $\text{C}$ is a penalty parameter, and the greater its value is, the heavier the penalty for misclassification. The first term in the objective function (2) is to increase the classification interval, which effectively controls the generalization ability of the model. The second term is the training error to reduce the experience risk.

To map the training data set to the high-dimensional space, a kernel function needs to be introduced; that is, the convex programming problem of Eq. (2) is transformed into a quadratic programming problem. The expected weight vector can be written as $\text{w} = \sum\limits_{i = 1}^{n} {(\alpha_{i}^{*} - \alpha_{i} )} {\mathbf{x}}_{{\mathbf{i}}}$, and finally, the analytical expression of the support vector machine regression function is as follows:

4 $$f\text(x) = \sum\limits_{i = 1}^{n} {(\alpha_{i} - \alpha_{i}^{*} } )K(\text{x},{\mathbf{x}}_{{\mathbf{i}}} ) + \text{b}$$

where $\alpha_{i}$ and $\alpha_{i}^{*}$ are the nonnegative Lagrange multipliers and $K(\text{x},{\mathbf{x}}_{{\mathbf{i}}} )$ is a kernel function satisfying the Mercer condition. The commonly used kernel functions mainly include the polynomial kernel function, Gaussian kernel function, radial basis function kernel function and sigmoid kernel function.

The input sample set data have different physical meanings and different dimensions and orders of magnitude, and it is necessary to normalize the original data before learning and training. The normalization method selected in this paper is the mapminmax function, and its normalization formula is:

5 $$\widehat{x} = 2*(\text{x} - \text{x}_{\min } )/(\text{x}_{\max } - \text{x}_{\min } ) - 1$$

where $\widehat{x}$ is the normalized data, $\text{x}$ is the input data, $\text{x}_{\max }$ and $\text{x}_{\min }$ are the maximum and minimum values of the input data, and the range of normalized data is between −1 and 1.

The libsvm toolbox in MATLAB software is used for SVM model learning and training, and the radial basis function is selected as the kernel function, that is, $\text{K}(\text{x}_{\text{i}}, \text{x}_{\text{j}} {) = \text{exp}}\left( { - \frac{{\left\| {\text{x}_{\text{i}}- \text{ x}_{\text{j}} } \right\|^{2} }}{{2\sigma^{2} }}} \right)$. The combination of grid search and k-fold cross validation is used to determine the best penalty factor ($\text{C}$) and kernel function parameters ($\sqrt 2 \sigma$), that is, the different combinations of penalty factor and kernel function parameters are selected to calculate the mean square errors obtained through training, and one group with the smallest mean square error is obtained as the optimal parameters.

**Figure 3:**
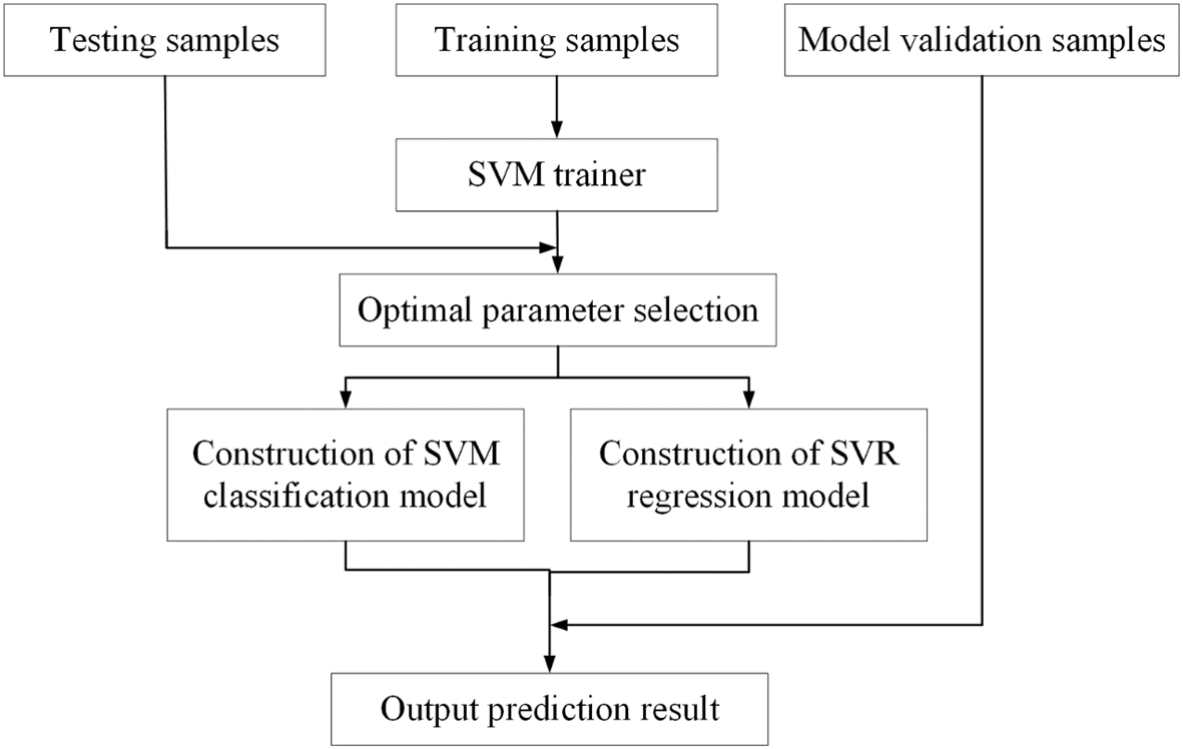
Flowchart of constructing the classification model and regression model by using the SVM method.

Figure 3 shows the flowchart of constructing the classification model and regression model by using the SVM method. The training samples are used for model training in the input data, the testing samples is used to determine the optimal model parameters, and the model validation samples are used to check the application effect of the constructed models.

### SVM classification model

Fluid identification using SVM is a multiclassification problem, but the SVM method initially solves two classification problems. Therefore, it is necessary to extend SVM method and construct a reasonable multiclassification coding scheme. At present, there are four main methods to construct SVM multiclassifiers: "one against one", "one against rest", "SVM decision tree" and "one-time solution method". When solving practical multiclassification problems, the "one-to-one" method has a better effect than other methods^29,30^. Therefore, this method is selected to construct an SVM multiclassifier in this paper, and the basic idea is that if there are class k data, class I data and class J data are selected to construct a classifier, where I < J, so k (k−1)/2 classifiers need to be trained. For class I and class J data, a two classification problem needs to be solved, and the voting method is used to solve this problem; that is, if the function judges that it belongs to class I, the number of votes of class I is increased by 1. Otherwise, the number of votes of class J is increased by 1, and the final output result is the class with the largest number of votes.

To build the SVM classification model for fluid identification, we must first determine the input logging data or parameters sensitive to the pore fluid. Considering that the study area is mainly conventional logging curves, nuclear magnetic resonance logs and array acoustic logs are not widely used in the whole area. Therefore, according to the characteristics of logging curve, the fluid identification factors sensitive to fluid type are selected as the input data, including ${(}{\text{PERM}}{/}\phi {)}^{{{1/2}}}$, $\text{D}_{{\text{R}}}$, $\text{QT}$, $\text{Rt}$, ${\Delta \text{SP}}$, $\text{R}_{{\text{wa}}}$ and $\text{R}_{{{\text{wa}\_\text{SP}}}}$, where ${(}{\text{PERM}}{/}\phi {)}^{{{1/2}}}$ is the comprehensive physical property index, which ${\text{PERM}}$ represents the permeability, and $\phi$ is the porosity of reservoir. $\text{QT}$ is the total hydrocarbon logging value, the greater the value, the greater the probability of possible oil and gas. $\text{Rt}$ is the resistivity logging value. The specific calculation methods of other parameters are as follows:

6 $${\Delta \text{SP} = }\frac{{\text{SP}_{{\text{Shale}}} { - \text{SP}}}}{{\text{SP}{}_{{\text{shale}}}-\text{ SP}_{{\text{sand}}} }}$$

where ${\Delta {\text{SP}}}$ is the relative amplitude of the spontaneous potential. When the salinity difference of formation water is small, the higher the oil saturation of the reservoir is, the smaller the ${\Delta \text{SP}}$ value; $\text{SP}$ is the spontaneous potential logging value; and $\text{SP}_{{\text{Shale}}}$ and $\text{SP}_{{\text{sand}}}$ are the spontaneous potential values of pure mudstone and pure sandstone, respectively.

7 $$\text{D}_{\text{R}} { = }\frac{{\text{AT90}}}{{\text{AT10}}} \times \frac{{\text{AT90}}}{{\text{AT20}}} \times \frac{{\text{AT90}}}{{\text{AT30}}} \times \frac{{\text{AT90}}}{{\text{AT60}}}$$

where $\text{D}_{\text{R}}$ is the resistivity difference parameter, and its value is related to the characteristics of mud invasion into permeable formation. The $\text{AT10}$, $\text{AT20}$, $\text{AT30}$, $\text{AT60}$ and $\text{AT90}$ are the resistivity logs of 10in, 20in, 30in, 60in, and 90in depth from the wellbore, respectively. In the target interval we studied, the permeability of the reservoir is poor, and the micro pores are relatively developed. For the fresh water mud, the oil layer is characterized by low invasion, while the water layer is characterized by high invasion. Therefore, the value of $\text{D}_{\text{R}}$ is large for the oil layer, while the value for the water layer is small^31,32^.

8 $$\text{R}_{{\text{wa}}} { = }\frac{{\text{R}_{\text{t}} *\phi^{\text{m}} }}{{\text{ab}}}{ }$$

where $\text{R}_{{\text{wa}}}$ is the apparent formation water resistivity calculated by the Archie formula when the reservoir water saturation is assumed to be 100%, $\text{m}$ is the cementation index, and $\text{a}$ and $\text{b}$ are the cementation indices.

9 $$\text{R}{}_{{{\text{wa}\_\text{SP}}}}{ = }\frac{{\text{R}_{{\text{mf}}} }}{{{10}^{{_{{^{{\text{U}_{{\text{ssp}}} \text{/K}}} }} }} }}$$

where $\text{R}{}_{{{\text{wa}\_\text{SP}}}}$ is the resistivity of the pure water layer calculated by spontaneous potential logging data. $\text{R}_{{\text{mf}}}$ is the resistivity of the mud filtrate, and $\text{U}_{{\text{SSP}}}$ is the static spontaneous potential value. $\text{K}$ is the diffusion adsorption electromotive force coefficient. In water-saturated layers, $\text{R}_{{\text{wa}}}$ is equal to or less than $\text{R}{}_{{\text{wa}\_\text{SP}}}$, and with the increase in reservoir oil saturation, $\text{R}_{{\text{wa}}}$ is higher than $\text{R}{}_{{{\text{wa}\_\text{SP}}}}$.

The output characteristics are represented by digital labels representing different fluid types, in which the number 2 represents the oil layer, the number 1 represents the oil–water layer, the number − 2 represents the water layer, and the number − 1 represents the dry layer. According to the oil test conclusion of the target interval in the study area, the input logging parameters are matched and combined with the numbers representing different pore fluid types to form the input training set of the model. To ensure the effectiveness and representativeness of the input training set, 204 training samples are selected in the study area, of which 185 are training sample sets and 19 are test sample sets. Table 2 shows the logging parameters and oil test results of these 19 test sample sets.

**Table 2:** The logging parameters and oil test results of these 19 test sample sets.

NO	Top (m)	Bottom (m)	${(\text{PERM}/}\phi {)}^{{{1/2}}}$	$\text{Dr}$	$\text{QT}$	$\text{Rt}$	${\Delta \text{SP}}$	$\text{R}_{{\text{wa}}}$	$\text{R}_{{\text{wa}\_\text{SP}}}$	Oil test results	
										Oil (t/d)	Water (m^3^/d)
1	2502.7	2503.5	0.231	0.621	21.161	146.661	0.675	2.364	0.258	33.32	0
2	2516.4	2740	0.170	2.344	1.863	119.823	0.702	1.348	0.327	31.96	0
3	2565	2571.3	0.274	1.648	9.201	61.624	0.697	1.146	0.274	8.0	0
4	2356.1	2360.5	0.139	1.014	0.53	16.735	0.657	0.294	0.248	27.12	0
5	2531.6	2533	0.178	1.755	1.131	61.118	0.770	0.627	0.184	13.0	0
6	2590	2595.8	0.272	0.814	0.483	45.347	0.770	0.666	0.246	7.88	0
7	2397.6	2401.8	0.166	1.087	2.409	32.619	0.760	0.369	0.229	11.9	0
8	2469.4	2472.9	0.170	0.947	1.281	9.353	0.852	0.215	0.272	4.34	10.7
9	2813.5	2816.2	0.216	1.492	0.945	13.018	0.713	0.241	0.236	4.68	2.5
10	2652.8	2656.8	0.359	0.878	2.545	13.015	0.858	0.253	0.155	1.56	10.6
11	2607.4	2609.8	0.216	1.492	0.945	13.018	0.713	0.236	0.241	11.22	5.6
12	2614.2	2618	0.496	0.687	2.713	7.585	0.776	0.248	0.156	4.86	6.5
13	2544.3	2548.7	0.297	0.992	1.179	208.161	0.797	2.750	0.292	5.44	6.9
14	2602	2605.3	0.151	1.055	1.06	43.868	0.812	0.463	0.244	6.58	10.9
15	2696.4	2698.8	0.756	0.707	0.375	4.898	0.76	0.174	0.294	0	12.2
16	2595	2600.5	0.144	0.670	0.313	8.736	0.833	0.149	0.142	0	33.6
17	2665	2667.2	0.583	0.390	3.643	6.832	0.793	0.166	0.197	0	19.8
18	2819.1	2822	0.208	0.838	2.254	7.603	0.864	0.183	0.098	0	11.0
19	2527	2529.2	0.080	0.303	2.552	104.618	0.421	0.692	0.290	0	0

**Figure 4:**
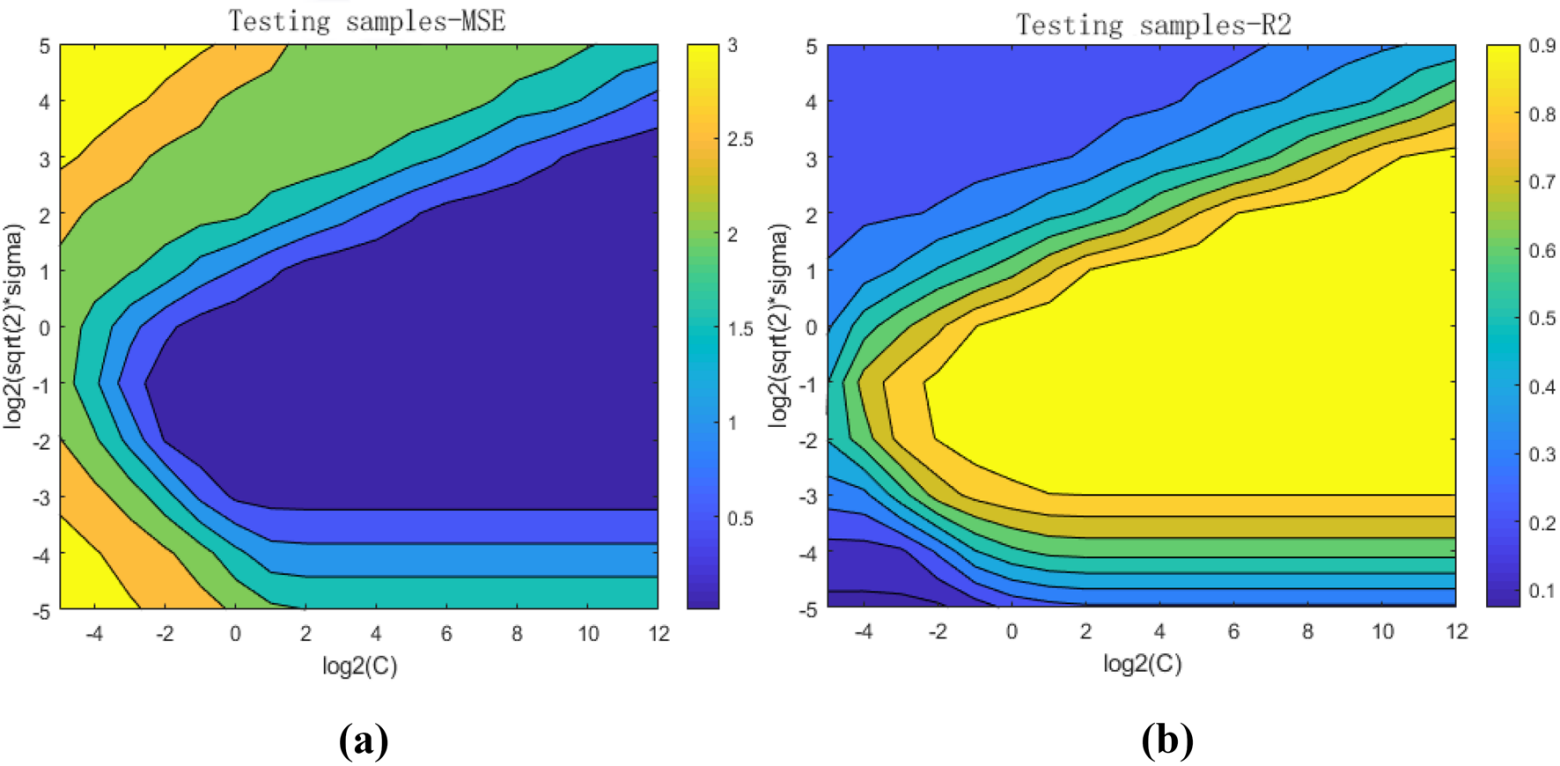
(**a**) The mean square errors of testing sample sets with different combinations of penalty factors and kernel function parameters, (**b**) the correlation coefficient of testing sample sets with different combinations of penalty factors and kernel function parameters.

Figure 4 shows the plan maps of the mean square error and correlation coefficient trained by the fourfold cross validation method under different $\text{C}$ and $\sqrt 2 \sigma$ parameter combinations. By looking for the penalty factor and kernel function parameters with the smallest mean square error and the highest correlation coefficient of 19 test sample sets, the optimal penalty factor and kernel function parameter combination of the classification model is C = 4096 and $\sqrt 2 \sigma$ = 2.

### SVR regression model

The permeability and water saturation of unconventional reservoirs are seriously affected by pore structure, and it is difficult to obtain these two parameters based on conventional logging curves. Therefore, the support vector regression (SVR) method is considered to construct the prediction model of reservoir permeability and water saturation. The idea of using SVR to build a reservoir parameter prediction model is the same as the basic process of the SVM classification model. which is to first select the optimal dataset with high correlation to the prediction target value as the input. The relationship between permeability, water saturation and logging curve is very complex. To determine the appropriate input training set, different logging data set combinations were used as the input training data, and the optimal input data set was selected by comparing the errors of the prediction model. The combination of different input logging data sets is shown in Table 3, including logging curves reflecting the reservoir lithology (ΔSP and ΔGR), reservoir physical properties (DEN, AC, CNL), reservoir electrical properties (RT), and reservoir porosity calculated by core calibration logging curve method (POR). And the optimal value of SVR model parameters ($\text{C}$ and $\sqrt 2 \sigma$) are still obtained by the fourfold cross validation method.

**Table 3:** The combination of different input logging data sets.

NO	Input logging data sets	Prediction parameter
Combination 1	DEN, AC, CNL	Permeability
Combination 2	RT, DEN, AC, CNL	
Combination 3	DEN, AC, CNL, ΔGR	
Combination 4	DEN, AC, CNL, ΔSP	
Combination 5	DEN, AC, CNL, ΔSP, POR	
Combination 1	DEN, AC, CNL	Water Saturation
Combination 2	RT, DEN, AC, CNL	
Combination 3	RT, DEN, AC, CNL, ΔGR	
Combination 4	RT, DEN, AC, CNL, ΔSP	
Combination 5	RT, DEN, AC, CNL, ΔSP, POR	

**Figure 5:**
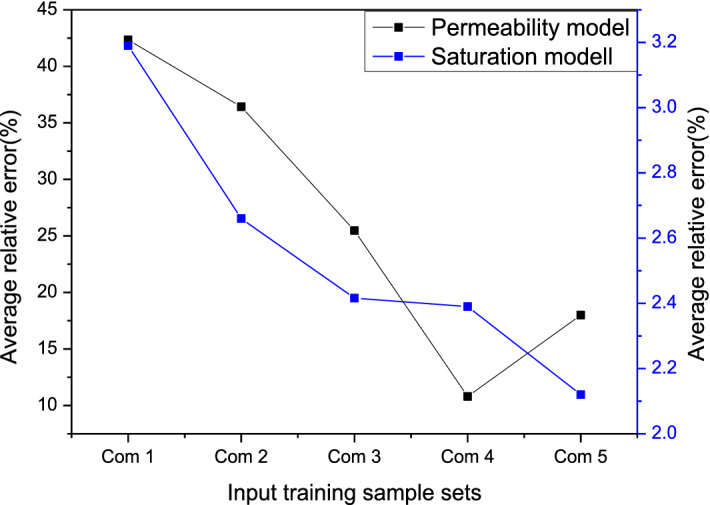
Characteristics of the average relative error by using different input data set combinations.

From 16 wells in the study area, approximately 252 reliable and representative closed coring data are selected to analyze the reservoir permeability and water saturation. And 50 samples are randomly selected for back judgment, and the optimal input training sample set combination is selected according to the average relative error of the back judgment results. Figure 5 shows the change in the average relative error of the regression permeability model and regression water saturation model when using different input data sets. Combination 4 has the smallest (10.7%) average relative error to predict reservoir permeability, which reflects that reservoir permeability is jointly affected by porosity and shale content. Adding porosity data cannot improve the accuracy. Combination 5 has the smallest (2.1%) average relative error to predict reservoir water saturation. From different average relative errors, the average relative error of the saturation regression model changes little from combination 2 to combination 5, basically floating up and down by 2%, which illustrates that the porosity data calculated by conventional methods can improve the accuracy, but it is not obvious, which also shows that the reservoir water saturation is mainly related to the electrical and comprehensive physical properties of the reservoir. Therefore, the optimal input training data set by the SVR regression permeability model is finally selected as combination 4, and the optimal input training data set by the SVR regression water saturation model is combination 5.

## Application effect analysis

### SVM classification model

To evaluate the reliability of the SVM classification model for fluid recognition, conventional fluid recognition method (cross plot of porosity and resistivity log), back propagation neural network (BP) method and radial basis function neural network (RBF) method were introduced for comparison. The input parameters of the BP and RBF neural network prediction model are the same as those of the SVM classification model. The optimal number of neuron layers of BP model is two layers, and the number of neurons in each layer is 12 and 14. The training function adopts the gradient descent adaptive learning rate function (traingdx function). The Gaussian function is selected as the basis function of RBF model, and the optimal Gaussian width of the training model is 0.1. Table 4 shows the comparison of fluid identification results of 19 test sample sets by using the SVM classification model, cross plot of porosity and resistivity log, BP model and RBF model. And the oil test results with only oil producing are resistivity low-contrast oil pays. It can be seen that the SVM classification model has the highest fluid identification accuracy (89.473%), followed by the RBF model (84.210%) and BP model (78.947%), and the conventional fluid recognition method has the lowest fluid identification accuracy (68.421%). This shows that using the SVM classification model to identify the resistivity low-contrast oil layer is effective and feasible. Moreover, compared with the commonly used artificial neural network algorithm (BP and RBF), the SVM classification model has certain advantages in solving the problem of small sample training, stronger generalization ability and better stability.

**Table 4:** Comparison of fluid identification results by different methods.

NO	Top (m)	Bottom (m)	Cross plot of Rt-Por		BP model		RBF model		SVM model		Oil test results	
			Fluid identification Result	Agreement	Fluid identification Result	Agreement	Fluid identification Result	Agreement	Fluid identification Result	Agreement	Oil (t/d)	Water (m^3^/d)
1	2502.7	2503.5	Oil layer	✓	Oil layer	✓	Oil layer	✓	Oil layer	✓	33.32	0
2	2516.4	2740	Oil layer	✓	Oil layer	✓	Oil layer	✓	Oil layer	✓	31.96	0
3	2565	2571.3	Oil layer	✓	Oil layer	✓	Oil layer	✓	Oil layer	✓	8.0	0
4	2356.1	2360.5	Oil layer	✓	Oil–water layer	×	Oil–water layer	×	Oil layer	✓	27.12	0
5	2531.6	2533	Oil layer	✓	Oil layer	✓	Oil layer	✓	Oil layer	✓	13.0	0
6	2590	2595.8	Oil–water layer	×	Oil–water layer	×	Oil–water layer	×	Oil layer	✓	7.88	0
7	2397.6	2401.8	Oil–water layer	×	Oil layer	✓	Oil layer	✓	Oil–water layer	×	11.9	0
8	2469.4	2472.9	Oil–water layer	✓	Oil layer	×	Oil layer	×	Oil–water layer	✓	4.34	10.7
9	2813.5	2816.2	Oil layer	×	Oil–water layer	✓	Oil–water layer	✓	Oil–water layer	✓	4.68	2.5
10	2652.8	2656.8	Oil layer	×	Oil–water layer	✓	Oil–water layer	✓	Oil–water layer	✓	1.56	10.6
11	2607.4	2609.8	Oil–water layer	✓	Oil–water layer	✓	Oil–water layer	✓	Oil–water layer	✓	11.22	5.6
12	2614.2	2618	Oil–water layer	✓	Oil–water layer	✓	Oil–water layer	✓	Oil–water layer	✓	4.86	6.5
13	2544.3	2548.7	Oil layer	×	Oil layer	×	Oil–water layer	✓	Oil–water layer	✓	5.44	6.9
14	2602	2605.3	Oil–water layer	✓	Oil–water layer	✓	Oil–water layer	✓	Oil–water layer	✓	6.58	10.9
15	2696.4	2698.8	Water layer	✓	Water layer	✓	Water layer	✓	Water layer	✓	0	12.2
16	2595	2600.5	Water layer	✓	Water layer	✓	Water layer	✓	Water layer	✓	0	33.6
17	2665	2667.2	Water layer	✓	Water layer	✓	Water layer	✓	Water layer	✓	0	19.8
18	2819.1	2822	Water layer	✓	Water layer	✓	Water layer	✓	Water layer	✓	0	11.0
19	2527	2529.2	Oil layer	×	Dry layer	✓	Dry layer	✓	Oil layer	×	0	0
Accuracy			68.421%(13/19)		78.947%(15/19)		84.210% (16/19)		89.473% (17/19)		/	

### SVR model

**Figure 6:**
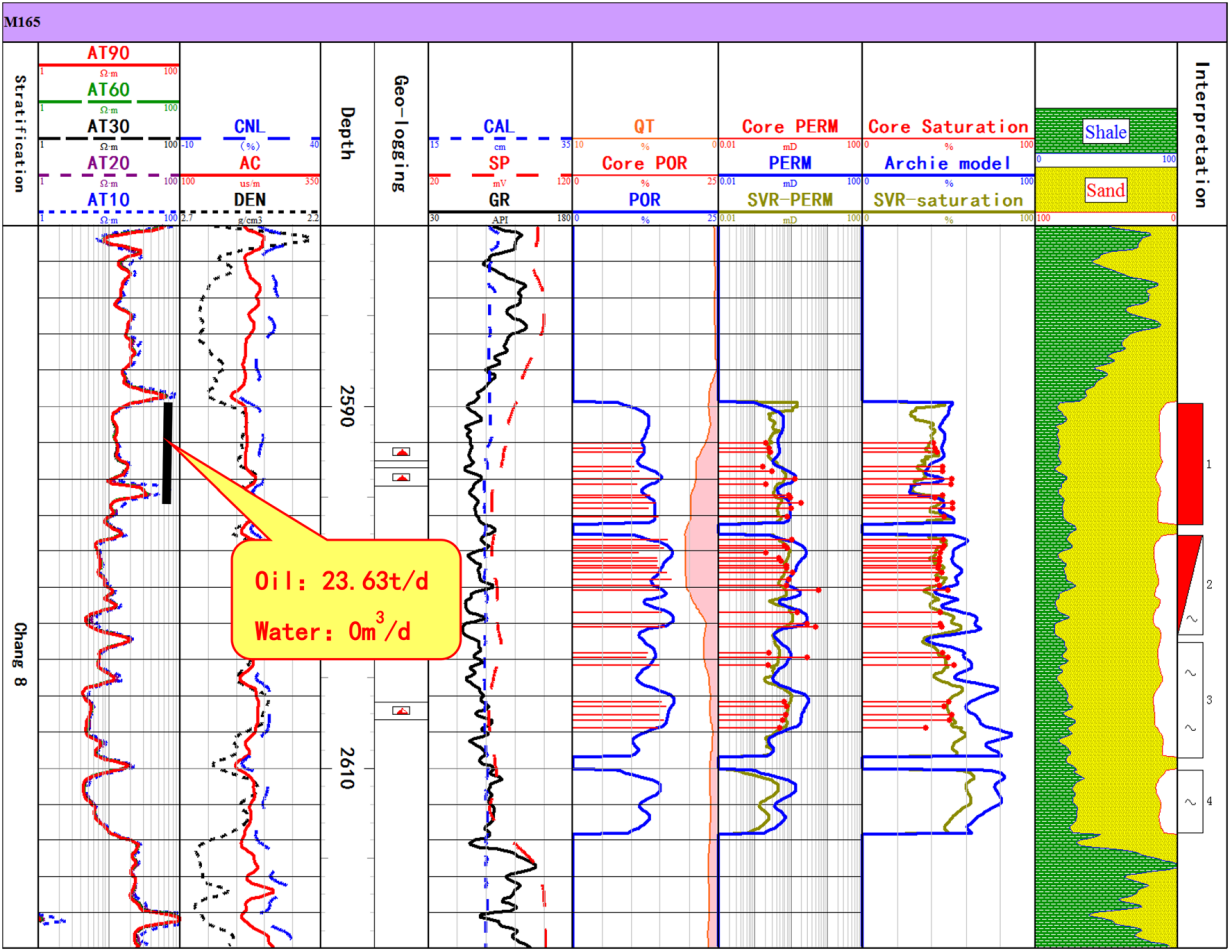
Comparison of reservoir permeability and saturation calculated by the SVR regression model and conventional method (Well M165).

Figure 6 is the log interpretation result of an oil production well (M165) with low resistivity, in which the testing interval is 2590–2596.5 m, and the average resistivity is about 12.6 Ω∙m. The 8th and 9th tracks in Fig. 6 are the calculation results of reservoir permeability and water saturation, respectively. The blue solid line in 8th track is the permeability calculated by the multiple logging curves regression of acoustic log and density log, and the yellow solid line is the permeability curve predicted by the SVR model. The blue solid line in 9th track is the saturation calculated by the Archie saturation model, and the parameters of Archie model are a = 1.0, b = 1.13, m = 1.99, n = 1.85 from petroelectric experiment of 16 cores. The yellow solid line in 9th track is the water saturation curve predicted by the SVR model. It can be seen that the reservoir parameters calculated by the SVR model are more consistent with the core analysis results.

**Figure 7:**
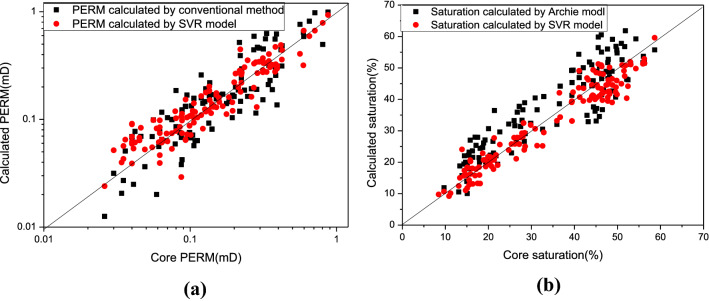
The comparison results of reservoir permeability (**a**) and water saturation (**b**) calculated by the SVR regression model and conventional method, respectively.

In addition, the calculated permeability and water saturation by using the SVR model and the conventional model are compared with the core analysis data of 129 sealed cores from 12 wells (Fig. 7). The results show that the average relative error of permeability calculated by the multiple logging curves regression model is 0.385, and the permeability predicted by the SVR model is 0.259. The average relative error of water saturation calculated by the Archie model is 0.188, while the saturation predicted by the SVR model is 0.097. This further verifies that the constructed SVR prediction model is feasible and effective.

### Discussion

Based on the support vector machine learning method, this paper constructs a classification model for resistivity low-contrast oil reservoir identification and SVR regression model for reservoir parameter prediction. Support vector machine learning method has the characteristics of low requirements for the number of training samples and not affected by local extremum and strong generalization ability, which makes it great advantages in solving complex practical problems such as nonlinear regression and classification compared with the classical neural network method. The application effect analysis also shows that the constructed model has a higher accuracy than the classical neural network prediction method and conventional logging interpretation model. However, it should be noted that during the process of model construction, the optimal input data set should be effectively selected. Therefore, in order to improve the application effect of SVM method in other similar areas, the logging response and reservoir characteristics of resistivity low-contrast oil pays should be analyzed to build an optimal input data sets.

## Conclutions

1. There is no obvious difference in physical and electrical properties between the resistivity low-contrast oil pay and water layer in the tight sandstone reservoir of the Chang 8 member in the Huanxian area, Ordos Basin. It is difficult to effectively identify and evaluate resistivity low-contrast oil pays by using conventional logging data, which seriously restricts the exploration progress and development benefits of oil resources in this area.
2. This study analyzed the relationship between the logging response and pore fluid to optimize the input training dataset. The SVM learning method was used to construct the SVM classification model and SVR regression model for fluid identification and reservoir parameter prediction.
3. The application results show that the SVM classification model has higher fluid identification accuracy, and the conventional fluid recognition method (cross plot of porosity and resistivity log) has the lowest fluid identification accuracy. The reservoir permeability and water saturation predicted by the SVR regression model are more consistent with the core analysis results, which proves that it is effective and feasible to interpret the resistivity low-contrast oil pays based on SVM method.

## References

[1] Pratama E, Ismail MS, Ridha S (2017). An integrated workflow to characterize and evaluate low resistivity pay and its phenomenon in a sandstone reservoir. J. Geophys. Eng..

[2] Bai Z, Tan MJ, Shi YJ (2022). An improved saturation evaluation method of Chang 8 tight sandstone reservoir in Longdong West area of Ordos Basin, China. Energy Exploration & Exploitation..

[3] Mode AW, Anyiam OA, Aghara IK (2015). Identification and petrophysical evaluation of thinly bedded low-resistivity pay reservoir in the Niger Delta. Arab. J. Geosci..

[4] Hakimov N, Zolfaghari A, Kalantari-Dahaghi A, Negahban S, Gunter G (2019). Pore-scale network modeling of microporosity in low-resistivity pay zones of carbonate reservoir. J. Nat. Gas Sci. Eng..

[5] Abuamarah BA, Nabawy BS, Shehata AM (2019). Integrated geological and petrophysical characterization of Oligocene deep marine unconventional poor to tight sandstone gas reservoir. Mar. Pet. Geol..

[6] Bai Z, Tan MJ, Li GR, Shi YJ (2019). Analysis of low-resistivity oil pay and fluid typing method of Chang 8(1) Member, Yanchang Formation in Huanxian area, Ordos Basin, China. J. Petrol. Sci. Eng..

[7] Shi WR, Zhang ZS, Huang ZS, Jiang S, Shen JC, Feng AG, Zhao HY, Xing J (2021). Investigation of the origin of low resistivity and methods for the calculation of gas saturation in shale gas reservoirs in the Fuling Area. Energy Fuels.

[8] Kadhim FS, Samsura A, Idris AK, Al-Dunainawi Y (2017). The use of artificial neural network to predict correlation of cementation factor to petrophysical properties in Yamamma formation. Int. J. Oil Gas Coal Technol..

[9] Shahriari M, Pardo D, Picon A, Galdran A, Delser J, Torres-Verdin C (2020). A deep learning approach to the inversion of borehole resistivity measurements. Comput. Geosci..

[10] Shehata AA, Osman OA, Nabawy BS (2021). Neural network application to petrophysical and lithofacies analysis based on multi-scale data: An integrate study using conventional well log, core and borehole image data. J. Nat. Gas Sci. Eng..

[11] Wang F, Yang XM, Zhang YH, Bian HY (2015). Application of multialgorithmic fusion methods to fluid identity in tight sand reservoir. Prog. Geophys..

[12] Liu D, Pan BZ, Zhou YF, Wang YB, Huang SH (2017). Fluid identification method based on high-resolution array induction logging using GA-SVM. Prog. Geophys..

[13] Guo YH, Pan BZ, Jiang BC, Liu SH, Fang CH, Li D (2015). Tight sandstone reservoir evaluation by the combination of three-water model and mathematical method in Sulige Area. Geophys. Prospect. Pet..

[14] Chen M, Peng ZL (2020). Study on low resistivity oil layer of Qudi oilfield based on BP neural network recognition. J. Ningxia Univ. (Natural Science Edition)..

[15] Singh Y, Nair RR, Singh H, Datta P, Jaiswal P, Dewangan P, Ramaprasad T (2016). Prediction of gas hydrate saturation throughout the seismic section in Krishna Godavari basin using multivariate linear regression and multilayer feed forward neural network approach. Arab. J. Geosci..

[16] Miah MI, Zendehboudi S, Ahmed S (2020). Log data-driven model and feature ranking for water saturation prediction using machine learning approach. J. Pet. Sci. Eng..

[17] Baouche R, Nabawy BS (2021). Permeability prediction in argillaceous sandstone reservoirs using fuzzy logic analysis: a case study of triassic sequences, Southern Hassi R’Mel Gas Field, Algeria. J. Afr. Earth Sci..

[18] Baneshi M, Behzadijo M, Schaffie M, Nezamabadi-Pour H (2013). Predicting log data by using artificial neural networks to approximate petrophysical parameters of formation. Pet. Sci. Technol..

[19] Roslin A, Esterle JS (2016). Electrofacies analysis for coal lithotype profiling based on high resolution wireline log data. Comput. Geosci..

[20] Yang H, Li SX, Liu XY (2013). Characteristics and resource prospects of tight oil and shale oil in Ordos Basin. Acta Pet. Sin..

[21] Fu JG, Dong GD, Zhong XP (2021). Research progress of petroleum geology and exploration technology in Ordos Basin. China Pet. Explor..

[22] Zhao JL, Yan B, Zhao JZ, Wang YP, Xu DC, Gao XL (2013). Reservoir characteristics of Chang 8 and single-well productivity prediction of M wellblock in Huanxian oil district, Longdong area. Oil Gas Geol..

[23] Chai GS, Shi YM, Du SH (2020). Sensitivity evaluation and influencing factors analysis of tight sandstone reservoirs: a case study of the Chang-8 reservoir in Yanchi Area of Ordos Basin. Acta Sci. Nat. Univ. Pekin..

[24] Liao P, Tang J, Pang G (2012). Reservoir characteristics and control factors of Chang 8_1_ of Yanchang formation of Triassic in Juyuan region of Ordos Basin. J. Miner. Petrol..

[25] Shi J, Lin YB, Zhao AB (2021). Diagenetic features and porosity dense evolution of Chang 8 tight sandstone reservoir in Hujianshan area, Ordos Basin. J. Pet. Explor. Prod..

[26] Yan WC, Sun JM, Zhang JY (2018). Studies of electrical properties of low-resistivity sandstone based on digital rock technology. J. Geophys. Eng..

[27] Bai Z, Tan MJ, Shi YJ, Li GR, Clark SM (2021). Studies of electrical properties of low-resistivity sandstone based on digital rock technology. J Pet. Explor. Prod. Technol..

[28] Vapink VN (1998). Statistical learning theory.

[29] Hsu CW, Lin CJ (2002). A comparision of methods of multiclass support vector machines. IEEE Trans. Neural Netw..

[30] Peng T, Zhang X (2007). Review on support vector machine and its applications in petroleum exploration and development. Prog. Explor. Geophys..

[31] Ding YJ, Shao ZW, Li QH (2009). A new method for reservoir liquid character evaluation with HDIL. Well Logging Technol..

[32] Salazar JM, Martin AF (2012). Rock quality assessment using the effect of mud-filtrate invasion on conflicting borehole resistivity measurements. Geophysics.

